# Cardiovascular Health Status And Genetic Risk In Survivors of Childhood Neuroblastoma and Nephroblastoma Treated With Doxorubicin: Protocol of the Pharmacogenetic Part of the LESS-Anthra Cross-Sectional Cohort Study

**DOI:** 10.2196/27898

**Published:** 2022-02-17

**Authors:** Oliver Zolk, Annika von dem Knesebeck, Norbert Graf, Thorsten Simon, Barbara Hero, Hashim Abdul-Khaliq, Mohamed Abd El Rahman, Claudia Spix, Benjamin Mayer, Susanne Elsner, Judith Gebauer, Thorsten Langer

**Affiliations:** 1 Institute of Clinical Pharmacology, Brandenburg Medical School (Theodor Fontane) Immanuel Klinik Rüdersdorf Rüdersdorf Germany; 2 Institute of Pharmacology of Natural Products & Clinical Pharmacology University of Ulm Ulm Germany; 3 Department of Pediatric Oncology and Hematology, Saarland University Homburg Germany; 4 Department of Pediatric Oncology and Hematology, Children's Hospital, University of Cologne Cologne Germany; 5 Department of Pediatric Cardiology, Saarland University Homburg Germany; 6 German Childhood Cancer Registry Mainz Germany; 7 Institute of Epidemiology and Medical Biometry University of Ulm Ulm Germany; 8 Institute for Social Medicine and Epidemiology, University of Lübeck Lübeck Germany; 9 Department of Internal Medicine I University Hospital of Schleswig-Holstein Lübeck Germany; 10 Department of Pediatric Oncology and Hematology, University Hospital for Children and Adolescents Lübeck Germany

**Keywords:** cardiotoxicity, anthracyclines, childhood cancer survivors, genetics, polymorphisms, cardiology, cardiac health, cancer, survivors, childhood, children, risk monitoring, genetics, cardiovascular health, pediatrics

## Abstract

**Background:**

In childhood cancer survivors (survival of 5 years or more after diagnosis), cardiac toxicity is the most common nonmalignant cause of death attributed to treatment-related consequences. Identifying patients at risk of developing late cardiac toxicity is therefore crucial to improving treatment outcomes. The use of genetic markers has been proposed, together with clinical risk factors, to predict individual risk of cardiac toxicity from cancer therapies, such as doxorubicin.

**Objective:**

The primary aim of this study is to evaluate the value of multimarker genetic testing for RARG rs2229774, UGT1A6 rs17863783, and SLC28A3 rs7853758 for predicting doxorubicin-induced cardiotoxicity. The secondary aim is to replicate previously described associations of candidate genetic markers with doxorubicin-induced cardiotoxicity. Moreover, we will evaluate the prevalence of cardiovascular dysfunction in childhood cancer survivors after neuroblastoma or nephroblastoma.

**Methods:**

This is the pharmacogenetic substudy of the research project Structural Optimization for Children With Cancer After Anthracycline Therapy (LESS-Anthra). We invited 2158 survivors of childhood neuroblastoma or nephroblastoma treated with doxorubicin according to the trial protocols of SIOP 9/GPOH, SIOP 93-01/GPOH, SIOP 2001/GPOH, NB 90, NB 97, or NB 2004 to participate in this prospective cross-sectional cohort study. The study participants underwent a cardiological examination and were asked to provide a blood or saliva sample for genotyping. The study participants' health statuses and cardiovascular diagnoses were recorded using a questionnaire completed by the cardiologist. Digital echocardiographic data were centrally evaluated to determine the contractile function parameters. Medical data on the tumor diagnosis and treatment protocol were taken from the study documentation. Survivors were screened for variants of several candidate genes by TaqMan genotyping.

**Results:**

This study includes 657 survivors treated with doxorubicin for childhood cancer, the largest German cohort assembled to date to investigate cardiovascular late effects. Data analyses are yet to be completed.

**Conclusions:**

This study will define the genetic risk related to 3 marker genes proposed in a pharmacogenetic guideline for risk assessment. Moreover, the results of this study will show the prevalence of cardiovascular dysfunction in survivors of pediatric neuroblastoma or nephroblastoma who were treated with doxorubicin. The results will help to improve primary treatment and follow-up care, thus reducing cardiovascular late effects in the growing population of childhood cancer survivors.

**Trial Registration:**

German Clinical Trials Register DRKS00015084; https://www.drks.de/drks_web/navigate.do?navigationId=trial.HTML&TRIAL_ID=DRKS00015084

**International Registered Report Identifier (IRRID):**

DERR1-10.2196/27898

## Introduction

Despite the emergence of new so-called targeted therapies, standard cytotoxic chemotherapies remain a cornerstone for treating various cancers. Anthracyclines, such as doxorubicin, daunorubicin, and epirubicin, are still among the most used chemotherapeutic agents. However, cardiotoxicity is a frequent complication with this class of anticancer drugs. In particular, childhood cancer survivors are at risk for cardiovascular complications, with a 15-fold increase in the relative risk of severe congestive heart failure compared to their noncancer siblings [1] and an 8-fold increase in mortality due to cardiovascular disease compared to the overall population [2,3]. Previous studies showed that 4.4%-42% of childhood survivors of acute lymphoblastic leukemia or nephroblastoma had progressive cardiac abnormalities several years after completing doxorubicin therapy [4-6].

Anthracycline-induced cardiomyopathy may develop during treatment or several years after completion of treatment and may include decreased left ventricular ejection fraction and signs and symptoms of congestive heart failure. Many risk factors for anthracycline-induced cardiotoxicity have been identified, most notably higher cumulative anthracycline doses and younger age. However, the optimal strategy for the prevention of anthracycline-induced cardiotoxicity is unclear. Currently, dexrazoxane is the only treatment for anthracycline cardioprotection approved by the United States Food and Drug Administration and European Medicines Agency. However, dexrazoxane is contraindicated in children and adolescents due to a 3-fold higher incidence of secondary malignancies in dexrazoxane-treated children compared with controls.

There is an additive or potentially synergistic increase in the risk of cardiomyopathy or cardiovascular death in anthracycline-treated patients who have received radiotherapy to the mediastinum or concomitant therapy with other known cardiotoxic agents, such as cyclophosphamide or vinca alkaloids [7,8]. Tukenova et al [7] reported that as little as 5 Gy of radiation to the heart increased the relative risk of cardiovascular disease mortality in childhood cancer survivors. New techniques in radiotherapy can increase cardiac protection without losing the efficacy of irradiation [9].

There is extensive interindividual variability in sensitivity to the cardiotoxic effects of anthracyclines. In some patients, cumulative doses of doxorubicin higher than 1000 mg/m^2^ cause no cardiomyopathy, whereas others develop cardiomyopathy at cumulative doses <200 mg/m^2^ [10]. Clinical and experimental studies have shown a considerable genetic contribution [11-13]. The heritability of anthracycline-induced cell toxicity has been estimated to be 35%-60% [11]. Together, the findings suggest that genetic factors play an important role in anthracycline-associated cardiotoxicity.

Candidate gene and genome-wide association studies have identified genetic variants associated with anthracycline-induced cardiotoxicity, including genetic polymorphisms in genes involved in anthracycline transport, metabolism, and anthracycline-induced oxidative stress. Based on their study results, Aminkeng et al [14] concluded that the evidence was strongest and most consistent for an association of *RARG* (retinoic acid receptor gamma) rs2229774, *SLC28A3* (solute carrier family 28 member 3) rs7853758, and *UGT1A6* (UDP glucuronosyltransferase family 1 member A6) rs17863783 variants with anthracycline-induced cardiotoxicity. Testing for these single nucleotide polymorphisms (SNPs) could improve discrimination between individuals at higher and lower risk of anthracycline-induced cardiotoxicity. Aminkeng et al [14] proposed the grading cardiotoxicity risk according to the *RARG* rs2229774, *UGT1A6* rs17863783, and *SLC28A3* rs7853758 genotype, as follows: patients carrying the *RARG* rs2229774 A or *UGT1A6* rs17863783 T risk variants should be considered at high risk of anthracycline-induced cardiotoxicity. The *SLC28A3* rs7853758 A allele was associated with a reduced risk of anthracycline-induced cardiotoxicity. For patients carrying the rs7853758 A protective variant who do not carry *RARG* rs2229774 or *UGT1A6* rs17863783 risk variants, classification into a lower cardiotoxicity risk group should be considered. All other patients should be considered at moderate genetic risk. An initial health economic evaluation of pharmacogenomic testing in patients treated for childhood cancer with anthracyclines suggests that information gained from the pharmacogenomic test could reduce mortality by approximately 17% and reduce costs by about 6% [15].

The primary aim of this study is to evaluate the importance of multimarker genetic testing for *RARG* rs2229774, *UGT1A6* rs17863783, and *SLC28A3* rs7853758 for predicting doxorubicin-induced cardiovascular dysfunction. We focus on these 3 markers because the only pharmacogenetic guideline published to date recommends their testing to stratify the individual cardiotoxic risk of anthracycline therapy. The secondary aim of this study is to determine the association of doxorubicin-induced cardiovascular toxicity with other candidate genes that have been previously described [13,16-19] or will soon be discovered, provided that our sample size has sufficient statistical power. To ensure that the population studied is as homogeneous as possible in terms of diagnoses and the type of anthracycline used, we focus on patients with childhood nephroblastoma or neuroblastoma. Another secondary objective is to investigate the prevalence of cardiovascular dysfunction in a population of children treated with doxorubicin.

## Methods

### Patients

This study is part of the LESS-Anthra cross-sectional cohort study initiated by the “late effects surveillance system” (LESS). As part of this study, former cancer patients were offered a standardized cardiological examination and were surveyed about their quality of life and health status. A goal of LESS-Anthra was to establish a biobank of blood samples to allow the investigation of genetic polymorphisms that may predispose an individual to doxorubicin-induced cardiomyopathy, which is the subject of this study protocol. The study was approved by the Ethics Committee of the University of Lübeck (14-182) and the Ethics Committee of the University of Ulm (239/17). Study participants were identified through LESS, a German multicenter active surveillance consortium studying late effects of cancer treatment in children, in collaboration with the German Childhood Cancer Registry. Patients who had survived childhood cancer for 5 years or more (since diagnosis) were eligible if they (1) were diagnosed with nephroblastoma or neuroblastoma between January 1990 and December 2012, (2) were diagnosed before the age of 18, (3) were assigned to a treatment protocol that included doxorubicin (intention-to-treat), and (4) had participated in one of the nephroblastoma trials SIOP (International Society of Paediatric Oncology) 9/GPOH (German Society for Pediatric Oncology and Hematology), SIOP 93-01/GPOH, or SIOP 2001/GPOH or neuroblastoma trials NB 90, NB 97, or NB 2004. In Germany, almost all patients diagnosed with nephroblastoma or neuroblastoma between 1990 and 2012 who were willing to participate in a trial were included in one of the above-mentioned studies. Patients selected for LESS-Anthra were contacted by the German Childhood Cancer Registry, which has access to current survivor addresses. A total of 2173 patients were identified, contacted by mail, and invited to participate in the study. Those who did not respond received a reminder letter. Patients unwilling to participate in the study had the opportunity to provide us with their reason for nonparticipation via a response form. Patients were included only after they (or their parents or legal guardians, if patients were under 18 years of age) had provided informed consent. For adolescents ≥16 years, their written consent was also required.

### Study Design

Patients who agreed to participate were asked to see a cardiologist within the next 12 months for a cardiologic follow-up examination, which included an electrocardiogram and transthoracic echocardiography. Patients had the choice of having the examination performed by their supervising cardiologist or a cardiologist recommended by the LESS-Anthra study group. Cardiologists had knowledge of treatment exposure but no knowledge of genotypes. The cardiologists were asked to send original echocardiographic recordings, together with a completed form summarizing the clinical and instrumental examinations results, to the cardiologic evaluation center (Clinic for Pediatric Cardiology at Saarland University Hospital, HA-K). The cardiologist also took a blood sample for pharmacogenetic analysis or a saliva sample at the patient's option. Biosamples were sent to the genetics center (Genotyping Laboratory, OZ).

### Collection of Medical Data

The following data were extracted from clinical trial registries: cancer diagnosis, age at diagnosis, sex, tumor location, tumor stage, study name and study arm, chemotherapy, anthracycline doses (doses of doxorubicin, per cycle and cumulatively, as defined in the study protocol for the study arm to which the patient was assigned), other cardiotoxic drugs (ie, drugs with cardiotoxic potential listed in the 2016 ESC position paper [20]), radiotherapy, irradiation field (mediastinal, lung, or left abdominal irradiation is considered a relevant cardiac risk factor [6,21]), irradiation doses of the primary tumor, relapse, and therapy (chemotherapy and irradiation) of recurrent tumor (Textbox 1).

**Assessment and variables recorded.**

**Cancer diagnosis and cancer treatment-related variables (obtained from study centers of the neuroblastoma trials (NB 90, NB 97, and NB 2004) and the nephroblastoma trials (SIOP 9/GPOH, SIOP 93-01/GPOH, and SIOP 2001/GPOH).**

- Cancer diagnosis, tumor location, tumor stage
- Age at diagnosis
- Sex
- Study name and study arm
- Chemotherapy including anthracycline doses (per cycle and cumulative)
- Other cardiotoxic drugs (the drugs listed in the 2016 ESC position paper were considered as potentially cardiotoxic drugs)
- Additional radiotherapy: irradiation field, irradiation doses of the primary tumor, and the field that included the heart (mediastinal, lung, left abdominal)
- Length of posttherapy interval
- Relapse and therapy (chemotherapy and irradiation) of the recurrent tumor
- Comorbidities

**Results of interview and clinical and physical examination (obtained at presentation to cardiologist).**

- Anthropometry: weight (kg) and height (cm)

**Systolic and diastolic blood pressure (mmHg) according to current guidelines for conventional office blood pressure measurement (ie, patients should be seated comfortably 5 min before measurements, at least 3 measurements 1-2 min apart, blood pressure is recorded as the average of the last 2 readings)**

- Heart rate (beats per minute)
- ECG findings: Cardiac arrhythmia with type (bradycardia, tachycardia, ventricular, or supraventricular); heart block findings with type (atrioventricular block with degree, complete right bundle branch block, or complete left bundle branch block); QTc prolongation (ms); signs of cardiac hypertrophy with type (right heart hypertrophy or left ventricular hypertrophy); other ECG pathologies
- Findings on clinical examination (edema, dyspnea at rest, cyanosis, hepatosplenomegaly, jugular venous congestion, or arrhythmia by auscultation).
- Diagnoses made by the cardiologist: Heart failure (right heart failure, left heart failure, or global heart failure; NYHA stage); cardiomyopathy (dilated, hypertrophic, restrictive, or other); arterial hypertension (primary hypertension or secondary hypertension); cardiac arrhythmia (type)
- The patient's expressed subjective resilience in everyday life (5 level rating: very good, good, average, poor, and very poor).
- The patient's expressed subjective ability to engage in sports activities (2 level rating: sports activity possible/not possible).

**Echocardiographic parameters (echocardiographic evaluation center)**

- Left ventricular size: Linear measurements; volume measurements; left ventricular mass calculations
- Left ventricular function assessment: Global systolic function parameters (fractional shortening or ejection fraction); global myocardial function assessed by Doppler-derived Tei index
- Left and right atrium area and volume measurements

### Cardiologic Examination

Cardiologists were asked to perform echocardiographic examinations according to the instructions provided. Table 1 shows the minimal digital acquisition protocol for transthoracic echocardiography. The echocardiographic recordings were centrally evaluated by a pediatric cardiologist in a standardized way and blinded for treatment exposure and genetic test results to minimize interinvestigator variability. In rare cases, in which parts of the echocardiography recordings were of insufficient quality, the corresponding data points were treated as missing values.

Additional information, such as clinical examination findings, weight, height, heart rate, blood pressure, and physical performance of the patient, was collected using a questionnaire (Table 1). This questionnaire was completed by the cardiologist and sent to the cardiological evaluation center.

**Table 1:** Minimal digital acquisition protocol for transthoracic echocardiography.

View		Data type
**Long-axis view, M-mode**		Still frame
	At the level of the tip of the posterior mitral valve leaflet (basal myocardial segment)	
**Four-chamber view (2D)**		Loop
	Left atrium and left ventricle, endocardium and myocardium, with mitral valve, left atrium shown with maximum area	
	Right atrium and right ventricle, endocardium and myocardium, with tricuspid valve	
**Short-axis view (2D)**		Loop
	Round section through left ventricle at mitral valve level, right ventricle incised	
**Two-chamber view (2D)**		Loop
	Left ventricle, endocardium and myocardium	
**Three-chamber view (2D)**		Loop
	Left atrium, left ventricle and aorta, endocardium and myocardium, with mitral valve, left atrium shown with maximum area	
**Pulsed-wave or continuous-wave Doppler**		Spectral doppler, still frame
	Mitral valve	
	Tricuspid valve	
	Aortic valve	
	Pulmonary valve	

### Outcomes

The primary endpoint was cardiac dysfunction, as diagnosed by the cardiologist or revealed by a central assessment of transthoracic echocardiography. Cardiac dysfunction is presumed when at least one of the following criteria is true:

1. Diagnosis by the cardiologist of heart failure or cardiomyopathy reported in the questionnaire.
2. Left ventricular ejection fraction reduced to <50%.
3. Shortening fraction reduced to <25%.
4. Relative wall thickness reduced to <0.22.
5. Percentage systolic thickening of the interventricular septum and left ventricular (LV) posterior wall reduced to <33%.
6. LV Tei index (myocardial performance index) >0.40.
7. Right atrial volume at end systole/body surface area >30 ml/m^2^.

The secondary endpoint is cardiovascular dysfunction, defined as the composite of arterial hypertension (diagnosed by the cardiologist and reported in the questionnaire or current blood pressure measured during the visit ≥140 mmHg systolic or ≥90 mmHg diastolic), cardiac arrhythmia (diagnosed by the cardiologist and reported in the questionnaire), and cardiac dysfunction (as defined above).

### Genotyping

Genomic DNA was isolated from EDTA blood samples with the QIAamp DNA Blood Kit (Qiagen). Saliva was collected with the Oragene DNA collection kit (DNA Genotec), and genomic DNA was extracted from saliva samples using the prepIT L2P reagent (DNA Genotec). DNA was quantified by Quant-iT PicoGreen assay (Invitrogen), according to the manufacturer’s protocols. Genomic DNA samples were genotyped for *RARG* rs2229774, *SLC28A3* rs7853758, and *UGT1A6* rs17863783 (Table 2) by TaqManSNP genotyping (Applied Biosystems), using the Type-it Fast SNP Probe PCR Kit (Qiagen) with predesigned primers and probes (Applied Biosystems).

Laboratory assistants were blinded to the case-control status of the patients genotyped in the study. To ensure the accuracy of all genotyping results, multiple positive and negative controls and replicate samples were included in all genotyping assays and plates. The concordance of genotype calls between replicate genotyped samples was 100%.

Genomic DNA will also be used to replicate the association of doxorubicin-related cardiovascular toxicity with other candidate genes that have been described [22] or that will be discovered in the future, provided our sample size is sufficient.

**Table 2:** Overview of the single nucleotide polymorphisms tested in LESS-Anthra.

Gene	Reference SNP^a^ cluster ID	Alleles (Ref^b^>Alt^c^)	Chromosome	MAF^d^ (1000 Genomes, European population)	Gene consequence	Amino acid/codon change
*RARG*	rs2229774	G>A	12	A=0.064	Missense variant	NP_000957.1: p.Ser427Leu
*SLC28A3*	rs7853758	G>A	9	A=0.137	Synonymous variant	Leu (CTG)>Leu [TTG]
*UGT1A6*	rs17863783	G>T	2	T=0.023	Synonymous variant	Val (GTG)>Val [GTT]

^a^SNP: single nucleotide polymorphism.

^b^Ref: reference allele.

^c^Alt: alternative allele.

^d^MAF: minor allele frequency.

### Estimation of Case Numbers for the Pharmacogenetic Studies

The appropriate case number was estimated based on a published study of the association of the nonsynonymous *RARG* gene variant p.Ser427Leu (rs2229774) with anthracycline-induced cardiomyopathy (combined analysis of the discovery and replication cohorts: odds ratio 4.7, 95% CI 2.7-8.3; *P*<.001) [12]. Assuming an odds ratio of 4.7, an allele frequency (controls) of 6.4%, an alpha error of 5%, power of 80%, a control: case ratio of 3:1, and a dropout rate of approximately 10%, at least 33 cases and 99 controls, will be required to replicate with sufficient statistical power the association of *RARG* rs2229774 with anthracycline-induced cardiomyopathy.

### Data Collection

For data collection, the electronic ObTiMA system ontology-based trial management application was used [23]. ObTiMA has been validated for clinical trials. Every correction made to the entered data is traceable. Only authorized persons have access to the program and the data. Data backups occur regularly and automatically. In ObTiMA, electronic case report forms for capturing all patient data have been defined for this study. All data were pseudonymized before entry into the database of ObTiMA and handled according to the European General Data Protection Regulation.

### Statistical Analysis

The *RARG* rs2229774, *SLC28A3* rs7853758, and *UGT1A6* rs17863783 SNPs were checked for deviations from Hardy-Weinberg equilibrium (HWE). Departure from HWE was defined as *P*-HWE <.01 (after Bonferroni correction of the nominal value of *P* set at .05) and tested by a χ^2^ test of goodness of fit between the observed and expected genotypes.

Our primary aim was to assess the value of multimarker genetic testing for *RARG* rs2229774, *UGT1A6* rs17863783, and *SLC28A3* rs7853758 for predicting doxorubicin-induced cardiovascular dysfunction. To address that aim, we considered the following 3 predictive models. Model 1 includes only clinical risk factors, such as sex, age, irradiation (mediastinal, lung, or left abdominal), the use of other cardiotoxic drugs, length of follow-up, and cumulative dose of doxorubicin. Model 2 includes only genetic profiling. For each individual, the genetic risk will be scored as suggested by the Canadian Pharmacogenomics Network for Drug Safety (CPNDS) Clinical Practice Recommendations Group [14], based on multimarker genetic testing for *RARG* rs2229774, *UGT1A6* rs17863783, and *SLC28A3* rs7853758. Patients carrying the *RARG* rs2229774 A or *UGT1A6* rs17863783 T risk variants will be classified as high genetic risk; patients carrying the *SLC28A3* rs7853758 A protective variant who do not carry *RARG* rs2229774 A or *UGT1A6* rs17863783 T alleles will be classified as low genetic risk; all other patients will be classified as moderate genetic risk (Table 3). Model 3 includes both clinical risk factors and genetic profiling. The independent contribution of each risk factor to the outcomes will be determined using multivariable logistic regression analysis. The predictive accuracy of the model will be assessed by the area under the receiver operating characteristic curve.

**Table 3:** Genetic risk scoring of anthracycline-associated cardiotoxicity as suggested by the Canadian Pharmacogenomics Network for Drug Safety Clinical Practice Recommendations Group [14].

Genetic risk^a^	RARG^b^ rs2229774	UGT1A6^c^ rs17863783	SLC28A3^d^ rs7853758
High	A|G or A|A	Any genotype	Any genotype
High	Any genotype	G|T or T|T	Any genotype
Moderate	G|G	G|G	G|G
Low	G|G	G|G	A|A or A|G

^a^Risk scoring depends on the combined evaluation of three genetic markers, the polymorphisms rs2229774 in *RARG*, rs17863783 in *UGT1A6*, and rs7853758 in *SCL28A3*.

^b^RARG: retinoic acid receptor gamma.

^c^UGT1A6: UDP glucuronosyltransferase family 1 member A6.

^d^SLC28A3: solute carrier family 28 member 3.

Our secondary aim is to replicate the previously described associations of candidate genetic markers with anthracycline-induced cardiotoxicity if our sample size has sufficient statistical power. Association of the candidate SNPs with the outcome will be performed using logistic regression, adjusting for sex; age; mediastinal, left abdominal, and lung irradiation; the use of other cardiotoxic drugs; length of follow-up; and cumulative dose of doxorubicin. The Benjamini-Hochberg False Discovery Rate will be used to account for multiple testing.

## Results

### Recruitment

**Figure 1:**
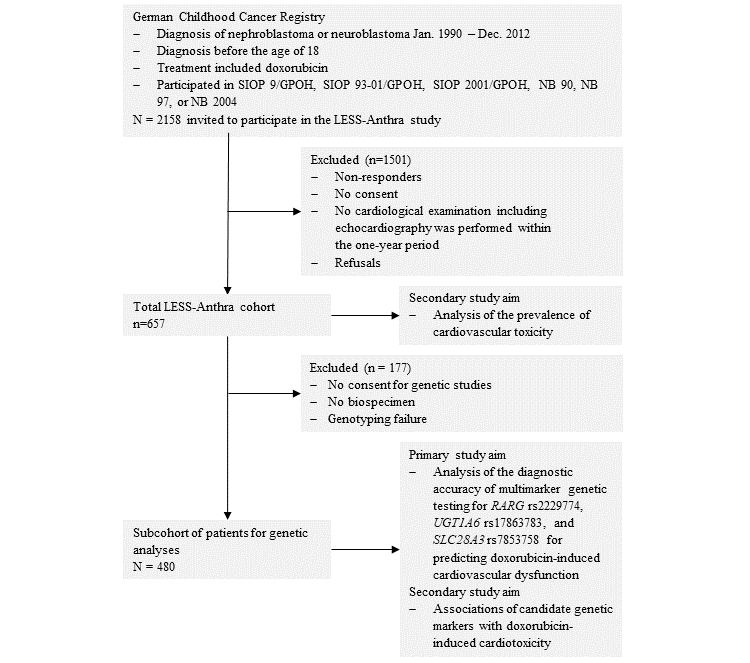
Flowchart of patients’ inclusion or exclusion.

This cohort study evaluates the risk of developing anthracycline-induced cardiotoxicity in survivors (ie, survival for 5 years or more after diagnosis) of pediatric nephroblastoma or neuroblastoma. A review of the German Childhood Cancer Registry database revealed 2158 eligible patients (943 nephroblastoma and 1215 neuroblastoma patients) who were invited by mail to participate in the study (Figure 1).

Patient recruitment started in June 2017 and was completed in September 2018. There were 657 patients (284 neuroblastoma and 373 nephroblastoma patients) who consented to participate in the LESS-Anthra study, and 480 (73%) of these patients provided a biospecimen for pharmacogenetic studies. Table 4 summarizes the baseline characteristics of participants and nonparticipants of LESS-Anthra.

**Table 4:** Comparison of baseline characteristics of participants and nonparticipants of the LESS-Anthra study.

	Patients invited for participation (n=2158)	Nonresponders, no consent, or refusal (n=1499)	Participants (n=657)
Age at diagnosis (years), median (range)	2.0 (0-17)	2.0 (0-17)	2.0 (0-17)
Follow-up time (years), median (range)	14.0 (5-27)	15.0 (5-27)	13.0 (5-27)
Proportion of male to female patients	1.0	0.9	1.2
Proportion of nephroblastoma to neuroblastoma patients	1.3	1.3	1.3

### Anthracycline Treatment

According to the respective protocol, and dependent on the stage and risk group, the cumulative dose of doxorubicin was 45-240 mg/m^2^ for neuroblastoma patients and 100-400 mg/m^2^ for nephroblastoma patients (Table 5).

**Table 5:** Per protocol cumulative dose of doxorubicin used in the neuroblastoma (NB90, NB97, and NB 2004) and nephroblastoma (SIOP 9/GPOH, SIOP 93-01/GPOH, and SIOP 2001/GPOH) trials.

Study and study arm		Doxorubicin cumulative dose (mg/m^2^)	Single-dose, duration of IV^a^ application
**NB90**			
	Stage 2 or stage 3A-B	120	Course N2: 60 mg/m^2^, 48h Course N3: 15 mg/m^2^, slow IV injection
	Stage 3C-D or stage 4	240	
	Stage 4S	60	
**NB97**			
	Standard risk group	120	Course N6: 30 mg/m^2^, 4h Course N4: 0.5 mg/kg, 30min
	High-risk group	180	
**NB 2004**			
	Observation group with tumor progression	45 or 180 (depending on further progression after first cycle)	Course N6: 30 mg/m^2^, 4h Course N4: 15 mg/m^2^, 30min
	Medium risk group	180	
	High-risk group	180	
**SIOP 9/GPOH**			
	Stage II, Stage III	250	50 mg/m^2^, 4h
	Stage IV, no metastasis after preoperative chemotherapy	400	
	Stage IV, no CR after preoperative chemotherapy	100	
**SIOP 93-01/GPOH**			
	Stage II or III and low or intermediate risk by histology	250	50 mg/m^2^, 4h
	Stage II or III and high risk by histology	300	
	Stage IV	400	
**SIOP 2001/GPOH**			
	Stage I, high risk	250	50 mg/m^2^, 4-6h
	Stage II or stage III, intermediate-risk, randomized regimen AVD	250	
	Stage II or stage III, high risk	300	
	Stage IV	300	

^a^IV: intravenous.

### Genotyping

We received biomaterial from 480 patients: 456 EDTA blood samples and 24 saliva samples. DNA was extracted and genotyped for *RARG* rs2229774, *SLC28A3* rs7853758, and *UGT1A6* rs17863783. Genotype frequencies for the candidate SNPs are summarized in Table 6. The call rate was 100% for all SNPs. All SNPs passed the HWE test at *P*>.01. Table 7 summarizes the numbers of patients in each genetic risk group, scored according to the CPNDS Clinical Practice Recommendations Group [14] and based on multimarker genetic testing for *RARG* rs2229774, *UGT1A6* rs17863783, and *SLC28A3* rs7853758.

**Table 6:** Genotype frequencies in the study cohort of neuroblastoma and nephroblastoma patients.

SNP^a^ and genotype		N	*P-*HWE^b^
*RARG*^c^ rs2229774			
	G/G	419	0.9604
	A/G	59	
	A/A	2	
*SLC28A3*^d^ rs7853758			
	G/G	355	0.848
	A/G	115	
	A/A	10	
*UGT1A6*^e^ rs17863783			
	G/G	467	0.764
	T/G	13	
	T/T	0	

^a^SNP: single nucleotide polymorphism.

^b^*P*-HWE: Hardy-Weinberg equilibrium χ^2^ test *P*-value

^c^RARG: retinoic acid receptor gamma.

^d^SLC28A3: solute carrier family 28 member 3.

^e^UGT1A6: UDP glucuronosyltransferase family 1 member A6.

**Table 7:** Genetic risk categories observed in our study population. Genetic risk scoring was performed according to the recommendations of the Canadian Pharmacogenomics Network for Drug Safety Clinical Practice Recommendations Group [14].

Genetic risk	Observed, n (%)
High	72 (15.0)
Moderate	301 (62.7)
Low	107 (22.3)
Total	480 (100)

### Data Collection

We are in the final phase of data collection. Currently, final plausibility and quality checks are being performed before the closure of the database.

## Discussion

The CPNDS Clinical Practice Recommendations Group has issued a level B recommendation (moderate evidence base: at least one high-quality study or multiple moderate-quality studies) that pharmacogenomic testing for the variants *RARG* rs2229774, *SLC28A3* rs7853758, and UGT1A6*4 rs17863783 should be performed in all pediatric cancer patients with an indication for doxorubicin or daunorubicin therapy to stratify their cardiovascular risk. The recommendation was based on a few studies investigating the separate effects of each of the genetic markers on anthracycline-induced cardiotoxicity. Additional studies are required to increase confidence in the genetic associations further.

This prospective cohort study is the first to evaluate the ability of combined testing for *RARG* rs2229774, *SLC28A3* rs7853758, and UGT1A6*4 rs17863783, as recommended in the pharmacogenetic guidelines, to correctly predict doxorubicin-related cardiotoxicity in a large cohort of patients with consistent diagnoses. Strengths of this study include the homogeneity of the cohort in terms of diagnoses and treatment protocols, the fact that no anthracyclines other than doxorubicin were used, and the large sample size. Because echocardiographic findings are subject to inter‐reader variability, the original echocardiographic recordings will be evaluated in a standardized manner by a single pediatric cardiologist, thereby reducing potential bias. The image acquisition protocol guided the site sonographers in performing the echocardiographic examinations according to the specific needs of our trial. All these methods were designed to improve the accuracy of echocardiographic findings. Nevertheless, different producers of echocardiography equipment and software and site-specific machine settings can still contribute to the variability of findings.

Unfortunately, there is no generally accepted definition of anthracycline-induced cardiotoxicity based on echocardiography [20,24-26]. Currently, the Cardio-Oncology and Imaging Working Group of the German Society for Pediatric Cardiology and Congenital Heart Defects is preparing a position paper in collaboration with the GPOH and the German Society for Cardiology-Cardiovascular Research. This paper will standardize the specific echocardiographic parameters and thresholds that should be used to define normal or abnormal parameters in childhood cancer survivors treated with anthracyclines. Therefore, because this issue is not yet conclusively resolved, we may need to adjust the outcome definitions in our study, depending on the results of the upcoming guidelines.

The overall participation rate in the LESS-Anthra study was only 30%, and not all LESS-Anthra participants were also willing to participate in the pharmacogenetic substudy. The participation rate is at the lower end of the range of figures reported in the literature [27]. We attempted to give as many eligible patients as possible the opportunity to participate, regardless of whether or not they were followed up closely. This strategy required the rather impersonal approach of study invitation and study consent by mail. Recruitment would likely be more successful if limited to patients in regular follow-up care who were invited and personally approached by the follow-up physician. Helligsoe et al [27] addressed study participation in childhood cancer survivors in clinical late-effect studies [27]. The authors analyzed 80 published studies originating from 16 cohorts, with a median follow-up of 6.0 years. They found that overall participation rates ranged from 27% to 100% and speculated that more personalized recruitment strategies could increase participation rates. Our preliminary results confirm the finding that time since diagnosis does not influence participation [27]. Age at diagnosis also does not have a measurable impact. Interestingly, in LESS-Anthra, slightly more male patients were in the responder group than nonresponders (Table 5).

Although study limitations, such as the variable follow-up time and the low participation rate, will have to be considered, our study will define for the first time the combined genetic risk related to three marker genes proposed for risk assessment in the CPNDS pharmacogenetic guidelines. Moreover, the results of this study will identify the prevalence of cardiovascular dysfunction among survivors of pediatric neuroblastoma or nephroblastoma treated with doxorubicin. The results will help to improve primary treatment and follow-up care to reduce cardiovascular late effects in the growing population of childhood cancer survivors.

## Abbreviations

CPNDS: Canadian Pharmacogenomics Network for Drug Safety
GPOH: German Society for Pediatric Oncology and Hematology
HWE: Hardy-Weinberg equilibrium
LESS: late effects surveillance system
LV: left ventricular
RARG: retinoic acid receptor gamma
SIOP: International Society of Paediatric Oncology
SLC28A3: solute carrier family 28 member 3
SNP: single nucleotide polymorphism
UGT1A6: UDP glucuronosyltransferase family 1 member A6
